# Habitual volar dislocation of the ulnar head with a locked distal radioulnar joint after distal radius fracture: A case report

**DOI:** 10.1097/MD.0000000000021343

**Published:** 2020-07-17

**Authors:** Yuji Tomori, Mitsuhiko Nanno, Shinro Takai

**Affiliations:** a Department of Orthopaedic Surgery, Nippon Medical School Musashi Kosugi Hospital, Kanagawa; b Department of Orthopaedic Surgery, Ukima Central Hospital; c Department of Orthopaedic Surgery, Nippon Medical School Hospital, Tokyo, Japan.

**Keywords:** case report, habitual volar dislocation, malunited distal radius fracture, TFCC injury, ulnar head

## Abstract

**Rationale:**

Habitual volar dislocation of the ulnar head with a locked distal radioulnar joint (DRUJ) is a rare complication after distal radius fracture. We present a case of habitual volar dislocation of the ulnar head in a woman with a dorsally displaced malunited distal radius fracture.

**Patient concerns:**

A 72-year-old woman presented with occasional painful locking of the forearm in full supination. She had fractured the left distal radius in a fall 6 months previously. The fracture had been treated non-surgically with wrist immobilization in a long and short arm cast for 6 weeks. Physical examination showed no swelling of the left hand. The wrist locked when the forearm was fully supinated, and the patient was not able to pronate her forearm without reducing the ulna by pressing the left ulna down toward the DRUJ. She experienced sharp pain during the reduction procedure and pronation of the left forearm. The affected wrist had 91% motion compared with the contralateral wrist.

**Diagnosis:**

Radiography and computed tomography showed 28° dorsally angulated malunion of the distal radius and ulnar head subluxation with respect to the radius. Magnetic resonance imaging revealed disruption of the ulnar-side triangular fibrocartilage complex (TFCC) from the ulna fovea.

**Interventions:**

The patient desired surgery to address the painful click during full supination of her left wrist and the limitations in her activities of daily living. Corrective osteotomy of the distal radius and arthroscopic repair of the ulnar-side tear of the TFCC were performed. The TFCC was arthroscopically repaired to the ulnar fovea to stabilize the DRUJ.

**Outcomes:**

At 1 year postoperatively, radiography showed complete union of the radius. The affected side had 97% wrist motion compared with the contralateral wrist and a full range of forearm rotation without pain or clicking. The grasp strength was 100% compared with the normal wrist.

**Lessons:**

Malunited distal radius fracture with concomitant TFCC injury can result in habitual volar dislocation of the ulnar head due to severely dorsally angulated malunion of the radius and avulsion of the ulnar-side TFCC from the ulna fovea. This condition required corrective osteotomy plus TFCC repair.

## Abstract 2

Supplemental Digital Content is available in the text

## 1 Introduction

As malunion of the distal radius with dorsal angulation occasionally results in deformity, wrist pain, and functional disability, surgical intervention is usually recommended for patients with severely displaced or malunited distal radius fractures with dorsal angulation.^[1–3]^ However, non-surgical treatment of distal radius fracture is an alternative option for older adult patients who do not wish to undergo surgery, as older adult patients do not usually complain about residual symptoms limiting the activities of daily life(ADL), even when residual radial deformity exists after fracture treatment.^[1,4,5]^ Nevertheless, non-surgical treatment of distal radius fracture occasionally causes complications that limit ADL, even in older adult patients.^[1]^ We present a rare case of habitual volar dislocation of the ulnar head and locking of the *distal radioulnar joint* (DRUJ) when the forearm was fully supinated in a 72-year-old woman with dorsally angulated malunion of a distal radius fracture.

## 2 Case presentation

A 72-year-old woman presented with painful locking of the DRUJ during full pronation of the forearm. She had sustained a left distal radius fracture due to a fall 6 months previously. The fracture had been treated non-surgically via wrist immobilization with a long- and short-arm cast for 6 weeks. Bone union of the distal radius was obtained, but there was persistent 28° dorsally angulated malunion of the distal radius. The range of motion of her left wrist recovered after treatment. However, she had a 6-month history of painful habitual volar dislocation of the left wrist and referred to our hospital. The patient was a housewife who did not participate in sports.

**Figure 1:**
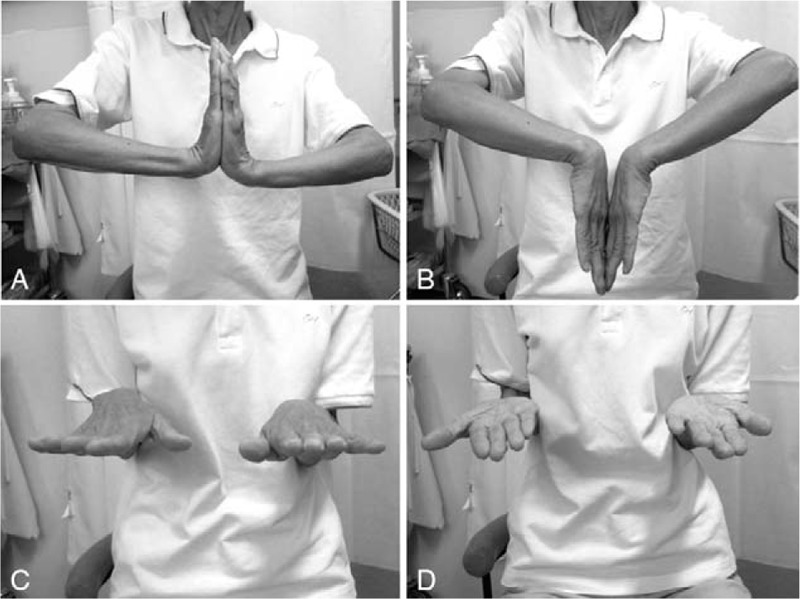
Initial clinical photographs showing habitual volar dislocation of the ulnar head in a 72-year-old woman. Wrist extension (A) and flexion (B). Forearm pronation (C) and supination (D).

**Figure 2:**
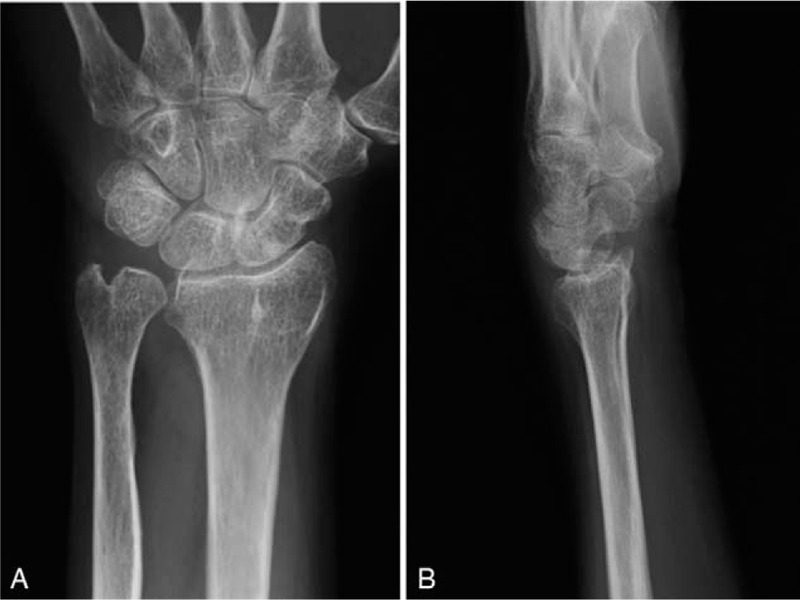
Radiographs of the left wrist at the time of presentation. Anteroposterior (A) and lateral (B) views. Although diastasis of the DRUJ is not seen on the anteroposterior view, there is slight volar displacement of the ulnar head in relation to the distal radius and carpal bones on the lateral view.

**Figure 3:**
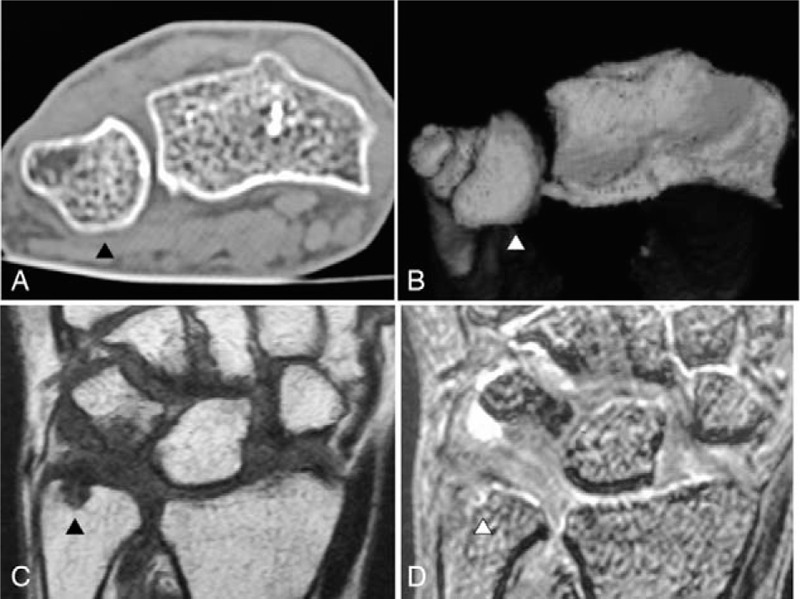
Imaging of the distal forearm. Select coronal cut of computed tomography (A) and three-dimensional computed tomography reconstruction (B) showing subluxation of the ulnar head to the anterior side of the sigmoid notch on the radius (arrowhead). Select coronal cut of magnetic resonance imaging of the wrist (C and D). The void in the ulnar head (arrowhead) indicates the disruption of the ulnar-side triangular fibrocartilage complex (TFCC) on a coronal proton density-weighted image (C). The high intensity region of the TFCC (arrowhead) indicates the avulsion of the ulnar-side TFCC on a coronal gradient recalled echo T2-weighted image (D).

Physical examination showed no swelling of the left hand. The wrist locked when the patient fully supinated her forearm, and she was not able to pronate her forearm without reducing the ulna by pressing it down toward the DRUJ (Supplementary Video 1 [The ulnar head was volarly dislocated and the wrist was locked when the patient fully supinated the forearm. Once the ulnar head was dislocated volarly at full supination of the forearm, the patient was not able to pronate her forearm without reducing the ulnar head by pressing down on her left ulna toward the distal radioulnar joint.]). The reduction procedure caused sharp pain during pronation of the left forearm. The left wrist showed 90° extension, 55° flexion, and full range of forearm rotation (Fig. 1A–D). The patient had 91% wrist range of motion compared with the contralateral wrist. The grasp strength was 7 kg, which was 64% of the grasp strength of the contralateral wrist. The modified Mayo wrist score^[6]^ (Table 1) was 25 (0-0-15-10; poor), and the patient-based clinical outcomes were a score of 68.1 in the disability of the arm, shoulder, and hand questionnaire,^[7]^ 101.5 in the Hand20 questionnaire,^[8]^ and 92.5 in the patient-rated wrist evaluation.^[9]^ Plain radiography showed that the distal radius was dorsally angulated at 28°, with malalignment of the DRUJ (Fig. 2A and B). Coronal computed tomography and three-dimensional computed tomography reconstruction of the distal forearm showed that the ulnar head was subluxated to the anterior side of the sigmoid notch on the radius (Fig. 3A and B). Magnetic resonance imaging revealed ulnar-side TFCC injury and avulsion of the ulnar fovea (Fig. 3C and D). Due to the painful click that occurred when the patient fully supinated her left wrist, and the limitations in her ADL, she desired surgery to treat the habitual volar dislocation of the ulna. The patient provided informed consent for surgery and gave written permission for her anonymized images to be used in the publication of this case.

**Table 1:**
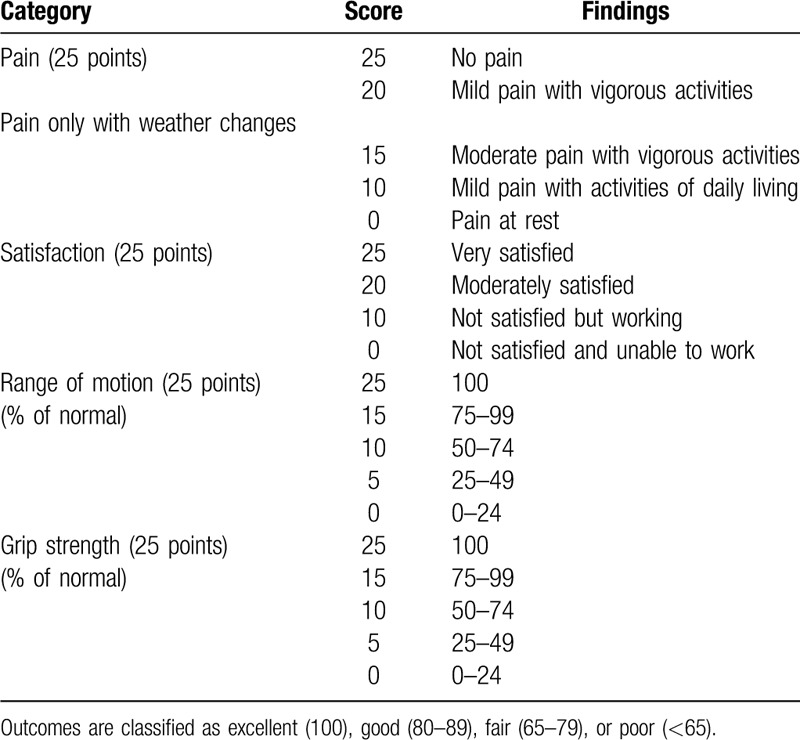
Modified Mayo wrist scoring system.

**Figure 4:**
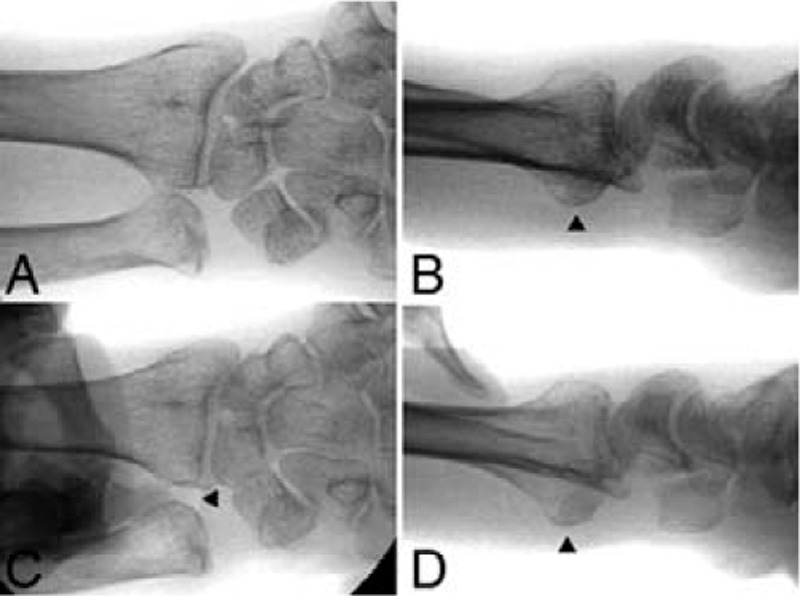
Perioperative radiographs of the left wrist. Anteroposterior (A), (C) and lateral (B), (D) views. Without stress, there is no diastasis of the DRUJ on the anteroposterior view (A), but the ulnar head is subluxated volar to the radius on the lateral view (B) (arrowhead). A manual stress traction test of the ulna shows marked diastasis of the distal radioulnar joint on the anteroposterior view (C) (arrowhead), and a volar press-down stress test of the ulnar head shows that the ulnar head is easily dislocated volarly to the radius on the lateral view (B) (arrowhead).

**Figure 5:**
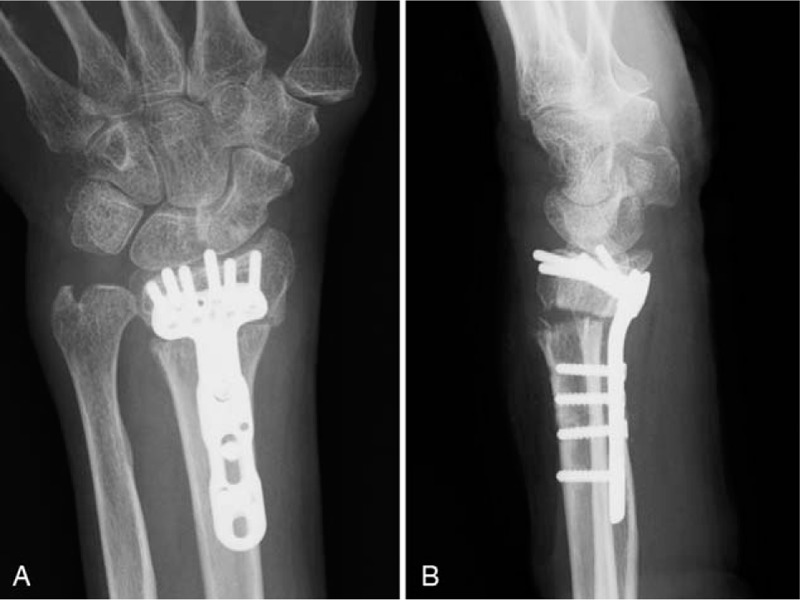
Postoperative radiographs of the left wrist. Anteroposterior (A) and lateral (B) views. The dorsal angulation of the distal radius is corrected and fixed with a volar locking plate.

Perioperative radiographs of the left wrist revealed that the ulnar head was subluxated volar to the radius on the lateral view, although diastasis of the DRUJ was not observed on the anteroposterior view (Fig. 4A and B). Under manual stress, there was marked diastasis of the DRUJ on the anteroposterior view, and the ulnar head was easily dislocated volar to the radius on the lateral view (Fig. 4C and D). Corrective osteotomy of the distal radius and arthroscopic repair of the ulnar-side tear of the TFCC were performed. The patient was placed in the supine position with the affected limb positioned to expose the surgical site, and an air tourniquet was applied. Malunion of the distal radius fracture was treated via open corrective osteotomy and internal fixation with a volar locking plate. Briefly, a longitudinal incision was made above the flexor carpi radialis (FCR), and the FCR was retracted radially. The tendon floor below the FCR was incised to expose the flexor pollicis longus. The flexor pollicis longus was retracted radially to expose the pronator quadratus. The pronator quadratus was divided and elevated to reveal the malunited site. The malunited radius was osteotomized, repositioned, and temporarily fixed with Kirschner wires under fluoroscopic guidance. The volar locking plate was placed, and the distal radius was fixed with locking screws and pins (Fig. 5A and B). After the corrective osteotomy of the distal radius, a manual stress test was performed. A volar press-down stress test of the ulnar head with respect to the radius revealed marked instability of the DRUJ. Thus, wrist arthroscopy was performed to evaluate TFCC injury. In the arthroscopic evaluation, a traction table was used to apply 3 kg of traction across the wrist. The 3 to 4 portal (between the extensor pollicis longus tendon and the extensor digitorum communis tendon) and a 4 to 5 portal (between the extensor digitorum communis tendon and the extensor digiti minimi tendon) were used to visualize and access the radiocarpal joint with a 2.3-mm 30° scope. Synovectomy was performed to obtain clear visualization of the articular surface of the distal radius and TFCC using a shaver system. The TFCC injury type was evaluated in accordance with Palmer's classification^[10,11]^ (Table 2). TFCC 1B injury (injury of the deep ligamentous portion) was diagnosed by indirect tests (positive trampoline test and positive hook test)^[12,13]^ using an arthroscopic probe (Supplementary Video 2 [Triangular fibrocartilage complex 1B injury {injury of the deep ligamentous portion} diagnosed by indirect tests {positive trampoline test and positive hook test} using an arthroscopic probe.]). In the arthroscopic repair, a 2-cm longitudinal incision was made on the ulnar side of the wrist just volar to the extensor carpi ulnaris (ECU). Blunt dissection was performed to expose the surface of the ulna while protecting the dorsal ulnar sensory nerve. Initially, the subluxated ulna was reduced to the DRUJ and maintained with temporary fixation with a 1.5-mm Kirschner wire. With the arthroscope in the 3 to 4 portal, a drill guide with a blunt rod was inserted through the 4 to 5 portal. The TFCC injury was treated via arthroscopic inside-out repair. Briefly, a suture-passing wire guide pin (AR-8914K; Arthrex, Naples, FL) was passed to the ulnocarpal joint through the 4 to 5 portal and pierced the articular disc proper of the TFCC via the ulnar side of the wrist joint. Then, the wire was drilled from the fovea of the ulna through to the ulnar edge of the ulnar head. A 2–0 FiberWire (Arthrex, Naples, FL) was threaded through the eye of the passing wire and advanced into the joint. The passing wire and the 2–0 FiberWire were pulled out to the ulnar edge of the ulna. Subsequently, under arthroscopy via the 3 to 4 portal, the cannula was moved to a position several millimeters to the volar side. Next, the passing wire was loaded into the cannula in the 4 to 5 portal, and the same procedures were repeated for the second pull-out suture on the dorsal side. Both ends of the FiberWire were sequentially rerouted so that the knot lay directly on the ulna with no interposed subcutaneous tissue, including potential nerve branches and the ECU. The FiberWire was tied rigidly to stabilize the ulna to the DRUJ, followed by the removal of the temporary Kirschner wire. After confirmation of the tension of the TFCC using an arthroscopic probe, the ulnar skin incision and arthroscopy portals were closed.

**Table 2:**
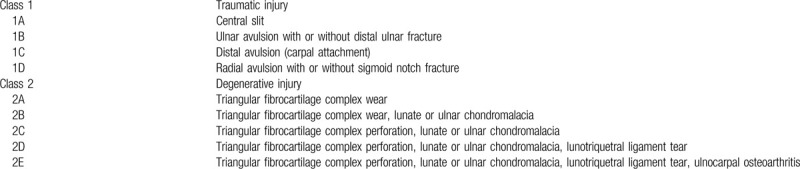
Palmer's classification of triangular fibrocartilage complex injures.

**Figure 6:**
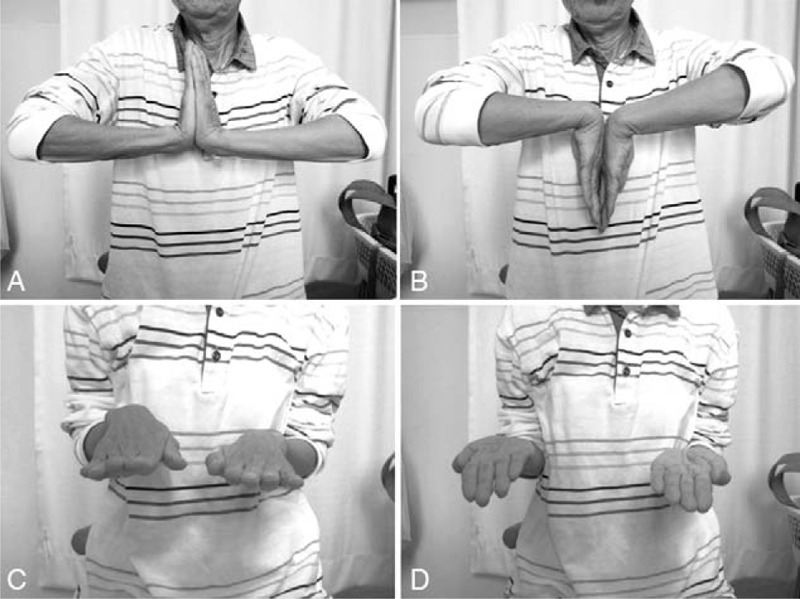
Clinical photographs at final follow-up 1 year postoperatively. Wrist extension (A) and flexion (B). Forearm pronation (C) and supination (D). The joint motion is 97% of normal without any pain or clicking, and with full forearm rotation.

**Figure 7:**
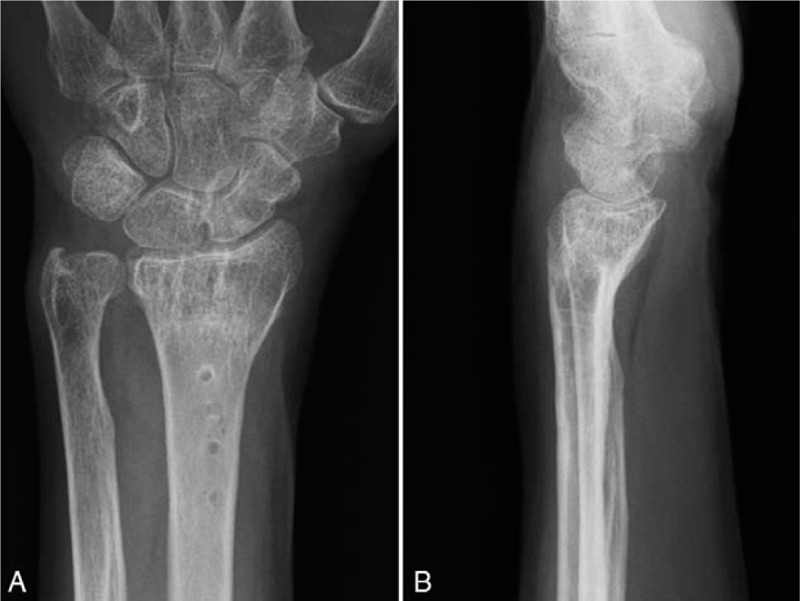
Radiographs at final follow-up 1 year postoperatively. (A) Anteroposterior and (B) lateral views show restored alignment of the distal radioulnar joint and corrected dorsal angulation of the distal radius.

Active extension and flexion exercises of the wrist were permitted from postoperative day 1. Three weeks postoperatively, the patient was encouraged to begin active rotation exercises of the forearm without restriction. At 6 months postoperatively, the plate was removed and arthroscopic evaluation of the radiocarpal joint revealed negative results for the trampoline test and hook test of the TFCC, showing recovery of the TFCC function (Supplementary Video 3 [6 months postoperatively, the plate was removed and the triangular fibrocartilage complex was arthroscopically evaluated. The trampoline sign was restored.]). At 1 year postoperatively, the wrist showed 80° extension, 70° flexion, and full range of forearm rotation (Fig. 6A–D). The joint motion reached 97% of normal without pain and/or clicking, and with full forearm rotation. The grasp strength was 14 kg, which was 100% compared with the contralateral wrist. The modified Mayo wrist score was 95 (25–25-20-25; excellent), and the patient-based clinical outcomes were a score of 0 in the disability of the arm, shoulder, and hand questionnaire, 2.5 in the Hand20 questionnaire, and 1.5 in the patient-rated wrist evaluation. The final follow-up radiographs of the left wrist at 1 year postoperatively showed that the alignment of the DRUJ had been restored and the dorsal angulation of the distal radius had been corrected (Fig. 7A and B).

## 3 Discussion

Dorsally angulated malunion of the distal radius is the most common complication after distal radius fracture.^[1,2,5,14]^ Although acute or chronic volar dislocation of the ulnar head with locking symptoms during forearm rotation have been reported,^[15–32]^ habitual volar dislocation of the ulnar head with a locked DRUJ is a rare condition that has only been reported in two cases in the English literature.^[28,33]^

The DRUJ enables pronation and supination of the forearm, and the head of the ulna translates volarly during supination and dorsally during pronation with respect to the distal radius.^[34]^ As the sigmoid notch of the distal radius is shallow and does not constrain the ulna during these movements, the stability of the DRUJ is enhanced by the TFCC, ulnar carpal ligaments, extensor retinaculum, pronator quadratus muscle, and interosseous membrane.^[34,35]^ The primary stabilizer of the DRUJ is the TFCC, which is composed of the dorsal and volar radio-ulnar ligaments, central articular disc, meniscus homolog, ECU sub-sheath, and ulnocarpal ligaments.^[10–12,34–36]^ The TFCC tethers the distal radius and ulna to stabilize the DRUJ, and restricts the displacement of the ulna with respect to the distal radius.^[10–12,34–37]^

A biomechanical cadaver study showed that dorsal angulation of the distal radius results in malalignment of the DRUJ with respect to the ulnar head, and volar instability of the ulna with respect to the distal radius.^[37–39]^ Incongruency of the DRUJ relative to the ulnar head occurs with increasing dorsal angulation of the distal radius, with the most dramatic change seen at more than 20° of dorsal angulation of the distal radius.^[39]^ Furthermore, volar displacement of the ulna with respect to the distal radius caused by dorsal angulation deformities is significantly larger with a sectioned TFCC compared with an intact TFCC, and dorsal angulation deformities with a sectioned TFCC move the ulna more volarly with respect to the distal radius during supination of the forearm.^[37,38]^ Thus, when dorsal angulation deformity of the distal radius occurs with an intact TFCC, the proximal radius shaft moves volarly with respect to the ulna to minimize the displacement at the distal end of the DRUJ when the tension in the TFCC increases.^[37,38]^ However, in patients with dorsally angulated malunion of the distal radius, ulnar-side TFCC injury results in volar translation of the ulna with respect to the distal radius.^[37,38]^

Although the magnitude of dorsal angulation is closely related to functional outcomes in younger patients, changes in DRUJ kinematics reportedly do not affect the functional outcomes in older adult patients who place minimal demands on their upper extremities.^[2,3]^ However, in our case, the dorsally angulated malunion of the distal radius resulted in malalignment of the DRUJ, which interfered with the containment of the ulnar head in the sigmoid notch. Moreover, the ulnar-side TFCC injury caused dysfunction of the primary stabilizer of the DRUJ and induced the habitual volar dislocation of the ulnar head, which resulted in painful locking of the DRUJ during full supination of the forearm.

Dorsal angulation deformity of the distal radius is usually acceptable in older adult patients.^[1,4,5]^ However, as severely dorsally angulated malunion of the radius with concomitant ulnar-side TFCC injury is a risk factor for volar dislocation of the distal ulna,^[37,38]^ it is essential to evaluate both the magnitude of osseous deformities of the distal radius and the integrity of the TFCC when managing acute or healed displaced fractures of the distal radius. Dorsal angulation of the distal radius with concomitant ulnar-side TFCC injury might require surgical treatment to prevent limitations of ADL due to instability of the DRUJ. In our case, corrective osteotomy of the malunited radius and arthroscopic ulnar-side TFCC repair achieved satisfactory outcomes for habitual volar dislocation of the distal ulna with a locked DRUJ after malunited distal radius fracture. Both restoration of the alignment of the DRUJ and stabilization of the DRUJ with repairment of ulnar-side TFCC injury are essential in cases with habitual volar dislocation of the ulnar head.

## 4 Conclusion

We reported a case of habitual volar dislocation of the ulnar head after non-surgical treatment of distal radius fracture. The habitual volar dislocation of the ulnar head was caused by malalignment of the DRUJ due to a dorsally angulated malunion of the distal radius, and ulnar-side TFCC injury in the left wrist. Corrective osteotomy of the malunited radius and arthroscopic ulnar-side TFCC repair achieved a satisfactory outcome.

## Acknowledgments

We thank Kelly Zammit, BVSc, from Edanz Group (www.edanzediting.com/ac) for editing a draft of this manuscript.

## Author contributions

**Conceptualization:** Yuji Tomori.

**Data curation:** Yuji Tomori.

**Investigation:** Yuji Tomori.

**Supervision:** Shinro Takai.

**Writing – original draft:** Yuji Tomori.

**Writing – review & editing:** Yuji Tomori, Mitsuhiko Nanno.

## Supplementary Material

Supplemental Digital Content

## Supplementary Material

Supplemental Digital Content

## Supplementary Material

Supplemental Digital Content
